# Marginal Fit of Single-Crown and Three-Unit Fixed Dental Prostheses Fabricated From Digital and Conventional Impressions: An In Vitro Cross-Sectional Study

**DOI:** 10.7759/cureus.73408

**Published:** 2024-11-10

**Authors:** Catherine N Maundu, Olivia A Osiro, James M Nyaga

**Affiliations:** 1 Conservative and Prosthetic Dentistry, Department of Dental Sciences, University of Nairobi Dental Hospital, Nairobi, KEN

**Keywords:** conventional dental impressions, digital dental impressions, fixed dental prostheses, intraoral scanners, marginal fit, polyether impression material

## Abstract

Introduction: With the current surge into digital dentistry, several options are available for clinicians, for example, when providing indirect restorations. There is a need for evidence on the quality of fixed dental prostheses (FDPs) fabricated using either digital or conventional impressions. This study aimed to evaluate the marginal fit of single-crown and three-unit FDP frameworks fabricated from digital and conventional impressions.

Materials and methods: Crown preparations were made on a maxillary typodont model (KaVo Dental GmbH, Biberach, Germany) on the right central incisor for a single-crown framework and the right first premolar and first molar for a three-unit framework to replace the second premolar. Four scanners (Dental Wings (DW, Straumann Group, Montreal, Canada), Carestream 3600 (CS, Carestream Dental, Atlanta, GA, USA), Medit i700 (M700, MEDIT Corp., Seoul, Republic of Korea), and Medit i500 (M500, MEDIT Corp.)) were used to record digital impressions of the preparations. Conventional impressions using polyether monophase impression material were also made, and stone casts were fabricated using high-strength stone and scanned using a laboratory scanner (Dental Wings, Straumann Group). Stereolithography files and computer-aided design and computer-aided manufacturing (CAD-CAM) were used to produce 50 zirconia FDPs (25 each of single crowns and three-unit frameworks). The marginal fit of the prostheses was determined by marginal gap measurements while seated on the typodont, a gap of ≤150µm being deemed acceptable. Results were summarized as means, standard deviations, medians, and interquartile ranges (IQRs). The independent t-test and one-way ANOVA followed by Tukey's post hoc test for means and Kruskal-Wallis test followed by Dunn’s post hoc test for medians were performed for hypothesis testing at α<0.05.

Results: The respective marginal gap measurements for single-crown and three-unit FDPs were 151.3±60.1µm and 153.9±50.1µm (polyether), 185.0±63.7µm and 224.2±81.7µm (DW), 177.1±81.3µm and 146.4±44.9µm (CS), 158.0±48.7µm and 184.3±86.2µm (M700), and 195.9±61.7µm and 202.8±71.1µm (M500). The marginal gap measurements of single crowns were significantly different among the five impression methods (F = 2.54, p = 0.042; χ^2^ = 14.68, p = 0.005) but not among the four digital methods (F = 1.83, p = 0.146), with the specific differences being between polyether and DW (p<0.01) and between polyether and M500 (p<0.001). The marginal gap measurements of the three-unit prostheses were significantly different among all five impression methods (F = 13.52, χ^2^ = 46.64, p<0.001) and the four digital methods (F = 12.32, p<0.001). The specific differences were between polyether and DW (p<0.001), M700 (p=0.02), and M500 (p<0.001), respectively; between CS and the other three digital methods (DW, p<0.001; M700, p=0.024; M500, p<0.001); and between DW and M700 (p=0.016).

Conclusion: Considering the means and standard deviations, all five impression techniques produced FDPs with acceptable marginal gap measurements. Significant differences were observed between conventional and digital impression techniques, with polyether and CS producing single-crown and three-unit FDPs having the least marginal gaps, respectively.

## Introduction

Technological innovations and new treatment protocols continue to challenge conventional approaches in dentistry [1]. For instance, in classical dental practice, dental impressions entail the use of a full or half-arched metal, silicone, or plastic impression tray. However, advancements have resulted in new techniques for obtaining digital impressions and manipulating digital data for diagnosis, treatment, and production of restorations. These technologies include digital cast scanners, intraoral digital impression-capture devices, cone beam computed tomography, three-dimensional (3D) printers, laser sintering units, and milling machines [2, 3]. Patients prefer these technologies, as they minimize the discomfort associated with traditional impressions [4]. Moreover, these modalities minimize chair and office time through improved efficiency, a reduced number of remakes, and lower laboratory bills. The transition to new dental technologies has led to improvements in the quality of oral care, better patient experience, and improved productivity and economics of dental practice [5].

Fixed dental prostheses (FDPs) and restorations can be made using conventional or digital techniques. Conventional metal-ceramic FDPs are fabricated using the lost-wax technique. However, this technique has several disadvantages, such as possible distortion of the wax patterns, defects in the cast metal, and complicated and time-consuming processing. New computer-aided design and computer-aided machining (CAD/CAM) materials and processes such as milling and 3D printing have countered these disadvantages because of their strength, insensitivity to thermal variations, rapid production, and dimensionally accurate nature [6, 7]. Digital impressions are generated either by scanning the stone cast using a laboratory scanner or through direct intraoral scanning of the prepared tooth [8, 9]. This digital workflow thus eliminates the need for the conventional impression and manual fabrication of the master cast stone model [10]. Accurate, aesthetic, and high-strength ceramic restorative materials are replacing the conventional porcelain layering technique [11-13].

One of the main factors affecting the longevity of a dental restoration is marginal adaptation, or how well it fits around the tooth [14, 15]. Improperly fitting FDPs can result in plaque accumulation, microleakage, and subsequent endodontic pathology. The marginal fit of a dental restoration is determined by various factors, such as the fabrication technique used, the number of units in the substructure, the tooth location and quality of preparation, the rigidity of the impression material, and the type and thickness of the luting agent [1, 15]. An accurate impression is fundamental to a successful outcome in prosthodontics. Impression and cast accuracy are influenced by the selection of impression materials, impression technique, operator skill, attention given to detail, and the production process of the working cast [9, 15]. Potential sources of error in conventional impressions may include improperly seated impression trays, dimensional changes in the impression material, deformation of the impression material, manipulation of the impression using a closed tray technique, and micromovement of the impression during attachment of the analog (for dental implants), while errors in digital impressions may arise from an unseated scanner, limitations in the accuracy of the digital intraoral scanner, errors introduced via the registration algorithm used to convert scan data to a digital impression and a digital impression to a digital model, and errors during milling of the working cast [16]. For both techniques, the presence of saliva and patient movements can influence the precision of the impression.

Studies have evaluated the fit of crowns and fixed partial dentures, but controversy still exists on the marginal fit of restorations manufactured using digital technologies. One study reported that the marginal fit of selective laser melting (SLM) was significantly inferior to that of the conventional lost-wax method [9], yet another found that the marginal fit of four-unit cobalt-chromium frameworks was superior when fabricated with SLM versus the conventional technique [6]. Furthermore, the effect of span length on the accuracy of restorations performed by conventional or digital techniques is unclear. An in vitro study to determine the impact of the scan extension and the starting quadrant while scanning concluded that this could influence the scanning trueness and precision among four intraoral scanners tested. Half arch scans of the tooth preparation site presented the highest trueness and precision, while the complete arch scans in which the scan started in the contralateral quadrant where the crown preparation was obtained showed the worst trueness and precision [17].

Long-span fixed partial dentures have been found to have a longer survival time than short-span fixed partial dentures, ideally because they have minimal torquing forces [18]. A 100% three-year survival rate [19], 85% 10-year survival rate [20], and 59% 20-year survival rate [21] have been reported for fixed partial dentures. However, they have also been associated with loss of retention and endodontic and periodontal problems as the main complications; thus, patient hygiene is an essential parameter during the implementation of FDPs [20, 22]. One investigation comparing prostheses fabricated from conventional or digital impressions reported that the digital impression approach using Encode abutments resulted in casts that were less accurate than casts generated from either conventional closed-tray or open-tray impressions [23]; however, another study showed that the mean marginal and internal fit values of the digital group were significantly smaller than those of the conventional group [24]. However, another study evaluating the accuracy of restoration analog positions using digital impressions of Encode healing abutments and conventional restoration-level impressions reported that both approaches resulted in slight inaccuracies in the restoration position, with the digital method associated with more inaccuracies than the conventional procedure, depending on the axis measured [25]. Evaluation of the time efficiency and fit of interim crowns fabricated using either a digital or a conventional workflow showed that the digital workflow required significantly less total fabrication time (laboratory and clinical) and resulted in a better fit, crown morphology, and occlusion than did the conventional workflow [26].

The importance of obtaining FDPs with a precise marginal and overall fit cannot be overemphasized, and the fabrication technique remains a critical determinant. The accuracy of the marginal fit of FDPs may be influenced by the quality of the intraoral or laboratory scanner as well as the span length of the prosthesis. Therefore, the purpose of this study was to evaluate the marginal fit determined by marginal gap measurements of single-crown and three-unit FDP frameworks fabricated using conventional and digital impression techniques. The null hypothesis under investigation was that there would be no difference in the marginal fit of these FDPs derived from the two techniques.

This article was previously posted to the Research Square preprint server on May 14, 2024.

## Materials and methods

Study design and setting

This was a laboratory-based analytical cross-sectional study conducted at Prime Dental Studio, a private dental laboratory in Nairobi, Kenya.

Sample size

The study sample size calculation was based on the formula for comparison of means as described by Kelsey et al. [27]. A total of 450 measurement points on 50 zirconia FDP frameworks were reproduced from three-tooth preparations for 25 crowns (single-tooth preparation) and 25 three-unit (two-tooth preparation) FDPs. Five impression techniques, one conventional and four digital, were used. A single crown had six measurable points, distal, mesial, and mid-mesial, both on the facial and the palatal aspects, while the three-unit FDPs had 12 points; that is, the same points on two retainers. For each impression technique, there were five specimens for the single crowns and the three-unit FDPs. The sample size is summarized in Table 1.

**Table 1:** Sample size calculation FDP: fixed dental prostheses

Type of prosthesis	Impression technique	Conventional (polyether)	Carestream	Dental wings	Medit i700	Medit i500	Total sample of points in a set of five specimens per prosthesis
	Specimen	Number of measured points					
Single-crown	1	6	6	6	6	6	30
	2	6	6	6	6	6	30
	3	6	6	6	6	6	30
	4	6	6	6	6	6	30
	5	6	6	6	6	6	30
Three-unit FDP	1	12	12	12	12	12	60
	2	12	12	12	12	12	60
	3	12	12	12	12	12	60
	4	12	12	12	12	12	60
	5	12	12	12	12	12	60
Total sample of points in a set of five specimens per technique		90	90	90	90	90	450

Data collection tools and techniques

**Figure 1:**
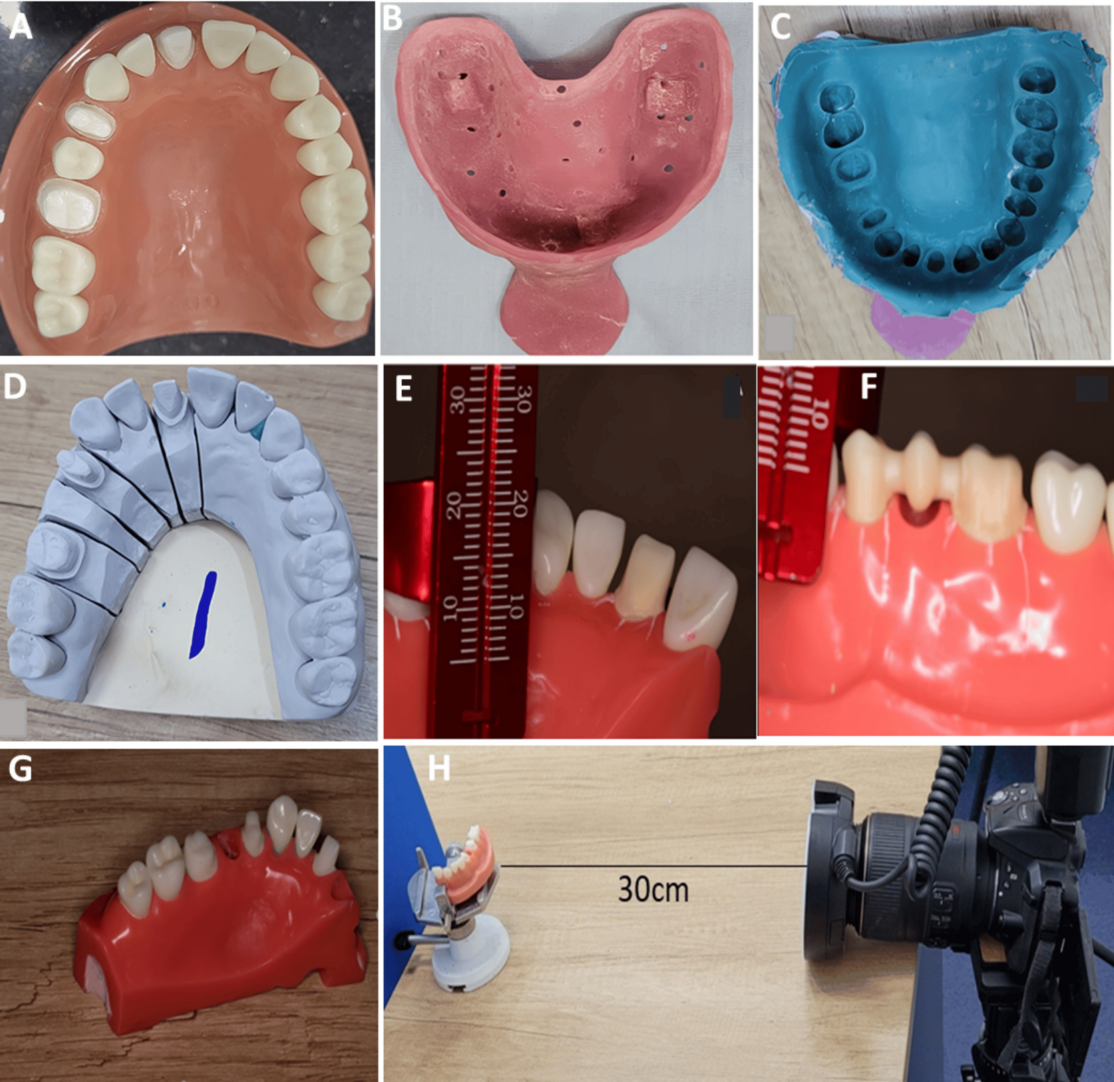
Illustration of data collection steps A: Typodont illustrating the tooth preparation prior to removal of the right second premolar and recording the impressions; B: Perforated, spaced master special tray fabricated from heat-polymerizing acrylic; C: Polyether impression; D: Resultant high-strength stone master cast; E: A single crown framework seated on a typodont model; F: A three-unit FDP framework seated on a typodont model. For both E and F, note the three markings on the mesial, mid, and distal aspects denoting the points at which marginal gap measurements were taken; G: Sectioned typodont model; H: Photography assembly

Data were collected in five steps: tooth preparation, impression making, prosthesis fabrication, photography, and image analysis, as illustrated in Figure 1. To minimize errors and ensure standardization of the procedures, the data were collected by one operator, the first author (CNM), who was calibrated by the third author (JMN), a prosthodontist.

Tooth preparation

Tooth preparation for a conventional all-ceramic FDP was conducted on a selected set of typodont teeth (KaVo Dental GmbH, Biberach, Germany). The mesiodistal tooth dimensions were 8.60 mm for the maxillary central incisor, 6.84 mm for the first premolar, 6.86 mm for the second premolar, and 10.1 mm for the first molar. Using a diamond shoulder bur on a high-speed handpiece, an approximately 2 mm shoulder margin was prepared around the maxillary right central incisor for a single crown, and the same was used for the maxillary right first premolar to the first permanent molar to replace a missing second premolar using a three-unit FDP (Figure 1A).

Impression making

In the digital group, the crowns and three-unit FDPs were fabricated using intraoral scanners and CAD-CAM technologies. Four digital scanners readily available in the Kenyan market were used according to the manufacturers’ instructions: Dental Wings (DW; Straumann Group, Montreal, Canada); Medit i500 (M500) and Medit i700 (M700; MEDIT Corp., Seoul, Republic of Korea); and Carestream 3600 (CS; Carestream Dental, Atlanta, GA, USA). Each preparation was scanned five times, and stereolithography (STL) files were produced from scanned images of the prepared teeth.

For the conventional group, impressions of the preparations for both the three-unit FDPs and single crowns were made using a special tray and polyether monophase material (Impregum Penta, 3M ESPE, St. Paul, MN, USA). A master special tray was fabricated from the standard model eight hours prior using heat polymerizing polymethylmethacrylate resin (Acropars® 200, Marlic Medical Industries Co., Tehran, Iran). The tray was spaced with two sheets of wax to achieve 2-3 mm spacer requirements and perforated, as shown in Figure 1B. Two impressions were taken using the monophase technique as recommended by the manufacturer. Each impression was used to fabricate two sets of five models cast in high-strength stone (Neelkanth Healthcare Pvt. Ltd., Jodhpur, India), one set being for the single-crown measurements and the other for the three-unit FDP measurements. An example of the polyether impression and stone model is shown in Figures 1C, 1D, respectively. Each stone model was then scanned using a laboratory scanner (DW, Straumann Group), and STL files were produced.

Prosthesis fabrication

Both sets of STL files from the four digital scans of the preparations on the typodont model and the stone models from polyether impressions were used to produce zirconia single-crown and three-unit FDP frameworks (1 Lava™ Plus Zirconia Disc 98S × 18 mm 3M, St. Paul, Minnesota, USA) through CAD-CAM. A zirconia milling machine (Aidite CMW-400, Qinhuangdao Technologies, Hebei, China) was used as per the manufacturer’s specifications. Prior to photography, the fitting surface of each prosthesis was inspected, and minor adjustments were made to ensure that the prosthesis was completely seated onto standardized original typodont models, which were preserved, as shown in Figures 1E, 1F.

Photography

After verifying that all the prostheses fit stably and were firm and immobile when pressed vertically on the corresponding prepared teeth in the typodont model, the model was separated into two sections, as shown in Figure 1G, to allow for photography of the facial and palatal aspects. A surveyor table was modified to be used as a jig to hold the master cast in place. Grooves were made on both ends of the sectioned model to enable a stable fit on the surveyor table. The master model was centered on the surveyor table, and the distance from the center of the table to the rim of the macro lens was set at 30 cm [28], as shown in Figure 1H. Three digital images of each of the fabricated prostheses were taken using a single-lens reflex camera (D5300; Nikon, Tokyo, Japan) with a macro lens (Nikkor AF-S 110 mm f/2.8G IF-ED; Nikon) in natural daylight between 10 a.m. and noon. The best image of the three that captured the facial, palatal, mesial, distal, midfacial, and midpalatal aspects of the prostheses was selected for image analysis.

Image analysis

The captured photographic images were uploaded onto a computer and magnified by a factor of 150% [28]. Computer on-screen caliper software (ImageJ, National Institutes of Health (NIH) and Laboratory for Optical and Computational Instrumentation (LOCI), the University of Wisconsin, WI, USA) was used to measure the vertical distance between the margin of the fitted FDP framework and the finish line. An endodontic ruler was placed against the models to calibrate and verify the measurements on the photos once uploaded to ImageJ software. To ensure reproducibility and validity, each of the six points was measured thrice on the selected photograph by CNM, with the examiner blinded to the previous measurements. The three measurements were then verified to be the same for the three readings and then recorded as one measurement for each site. This was repeated at six sites for the five models for each impression technique and FDP type, as described in the sample size in Table 1. Every fifth model was measured by a second examiner, JMN, and the kappa interexaminer reliability score was determined (r = 0.979, p<0.001).

Data analysis

The data were summarized using Microsoft Excel spreadsheets (Microsoft Corp., Redmond, WA, USA) and analyzed using STATA, version 16 (StataCorp LLC, College Station, TX, USA). Descriptive summaries included means, standard deviations (SDs), medians, and interquartile ranges (IQRs). The independent sample t-test and one-way ANOVA followed by Tukey's post hoc test for means and Kruskal-Wallis test followed by Dunn’s post hoc test for medians were used for hypothesis testing to analyze differences in the marginal fit determined by marginal gap measurements of FDP frameworks made from conventional and digital impression techniques at α = 0.05.

## Results

Within the context of this study, a marginal fit determined by marginal gap measurements of 150µm or less was deemed acceptable from a clinical standpoint.

**Figure 2:**
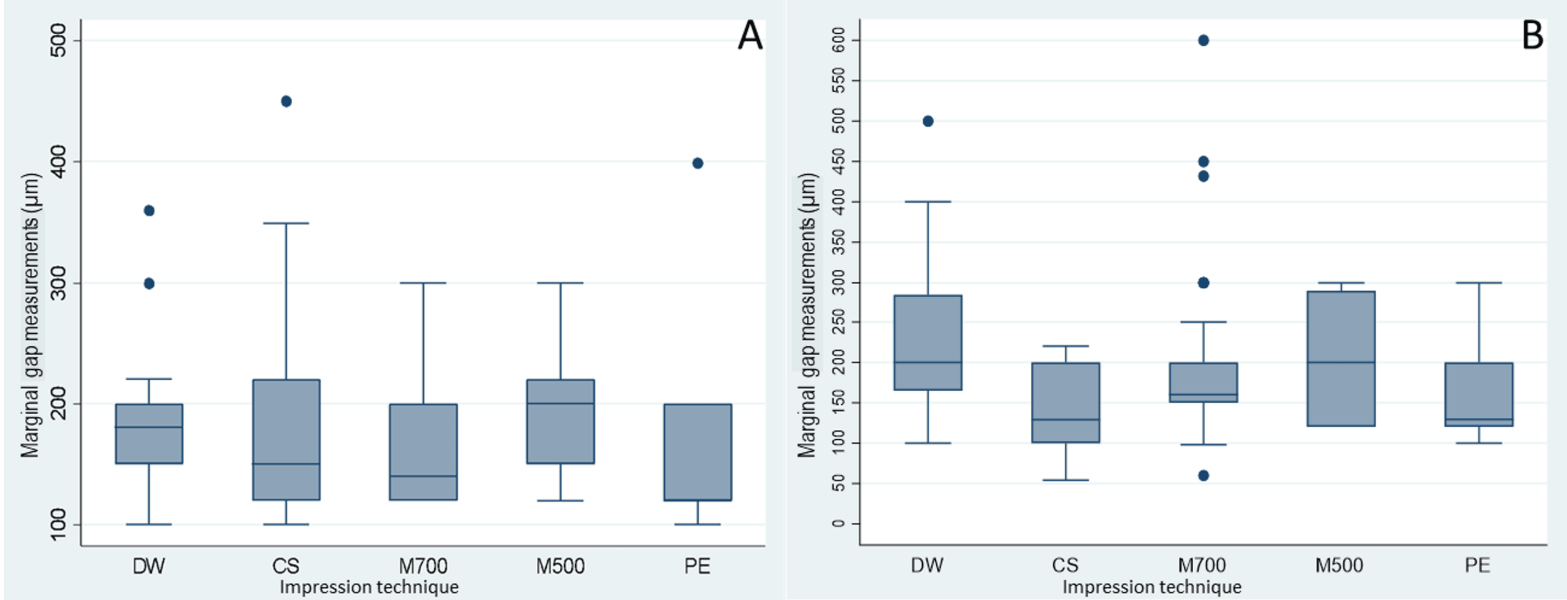
Summary of marginal fit determined by median marginal gap measurements of FDPs (A: single-crown; B: three-unit prosthesis) fabricated using conventional and digital impressions on models FDP: fixed dental prostheses

Table 2 and Figure 2 summarize the marginal fit determined by mean and median marginal gap measurements of FDP frameworks fabricated from conventional and digital impressions. The mean marginal gap measurements of single crowns ranged between 151.3±60.1µm for polyether impressions and 195.9±61.7µm for M500 digital impressions. For three-unit FDPs, the mean marginal gap measurements ranged between 146.4±44.9µm for CS digital impressions and 224.2±81.7µm for DW digital impressions.

**Table 2:** Summary of the marginal fit determined by mean marginal gap measurements of FDPs (single-crown and three-unit FDP frameworks) fabricated using polyether and four digital impressions on models (SD in parenthesis) FDP: fixed dental prostheses; SD: standard deviation

Impression method	Crown type	Site-specific marginal gap measurements (µm)						Overall marginal gap (µm)
		Distal		Mid-mesial		Mesial		
		Facial	Palatal	Facial	Palatal	Facial	Palatal	
Conventional impression (PE)	Single n=30	199.8 (122.1)	124 (16.7)	184 (35.8)	152 (43.8)	120 (0)	127.8 (25.8)	151.3 (60.1)
	Multiple n=60	120 (0)	164 (53.6)	110 (10.5)	159.5 (35.4)	165 (65.2)	205 (36.9)	153.9 (50.1)
Dental Wings scanner (DW)	Single n=30	154 (13.4)	180 (83.7)	176 (45.6)	180 (30.8)	224 (125.2)	196 (8.9)	185 (63.7)
	Multiple n=60	173 (33.7)	260 (69.9)	199 (43.8)	254 (43)	267 (132)	192 (85.5)	224.2 (81.7)
Carestream scanner (CS)	Single n=30	132 (16.4)	157.4 (41.4)	271.6 (101.3)	249.8 (70.4)	114 (21.9)	138 (50.2)	177.1 (81.3)
	Multiple n=60	138 (34.3)	189 (36.7)	174.4 (34.4)	125.7 (30.9)	105 (15.8)	146.4 (54.5)	146.4 (44.9)
Medit i700 scanner (M700)	Single n=30	132 (11)	126 (13.4)	140 (0)	141.8 (24.8)	200 (0)	208 (90.1)	158 (48.7)
	Multiple n=60	177 (21.1)	230 (153.8)	215 (85.1)	192.2 (87.8)	152 (44.9)	139.8 (28.2)	184.3 (86.2)
Medit i500 scanner (M500)	Single n=30	215.8 (81.1)	214 (79.9)	208 (90.1)	176 (51.8)	161.8 (28.0)	200 (0)	195.9 (61.7)
	Multiple n=60	176 (39.8)	281 (32.1)	300 (0)	168 (36.1)	123 (9.5)	169 (37.2)	202.8 (71.1)

As shown in Figure 2A, for single crowns, the lowest median marginal gaps were recorded for polyether impressions (120µm, IQR 120-200µm), while the highest median marginal gaps were recorded for M500 digital impressions (200µm, IQR 150-220µm). As shown in Figure 2B, for three-unit FDPs, the lowest median marginal gaps were recorded for polyether impressions (129.5µm, IQR 120-200µm) and CS digital impressions (129µm, IQR 100-200µm), while the highest median marginal gaps were recorded for DW digital impressions (200 µm, IQR 165-285µm) and M500 digital impressions (200µm, IQR 120-290µm).

Table 3 shows the difference in marginal fit determined by the mean and median marginal gap measurements of FDPs fabricated from conventional and digital impressions. One-way ANOVA and the Kruskal-Wallis test showed significant differences in the average marginal fit between impression methods for both single-crown (p<0.05) and multiple-crown (p<0.001) prostheses. Moreover, Dunn’s post hoc test revealed significant differences between conventional impression and DW and between PE and M500 for both single crowns and three-unit FDPs.

**Table 3:** Summary of differences in the mean and median marginal gap measurements of FDP frameworks fabricated from conventional and digital impressions FDP: fixed dental prostheses; DW: Dental Wings; CS: Carestream 3600; M700: Medit i700; M500: Medit i500; PE: conventional impression

Crown type	Impression method and measurements (µm, sd in parenthesis)		t value	p-value	95% CI	ANOVA F statistic (p-value)	Kruskall -Wallis χ^2 ^F statistic (p-value)	Dunn’s post hoc p-value
Single-crown (n=150)	DW	PE						
	185 (63.7)	151.3 (60.1)	2.71	0.039	1.7, 65.7	2.54 (0.042)	14.68 (0.005)	<0.01
	CS	PE						
	177.1 (81.3)	151.3 (60.1)	1.40	0.166	-11.6, 62.8			
	M700	PE						
	157.9 (48.7)	151.3 (60.1)	0.47	0.637	-21.6, 34.9			
	M500	PE						
	195.9 (61.7)	151.3 (60.1)	2.84	0.006	13.2, 76.1			<0.001
Three-unit FDP (n=300)	DW	PE						
	224.2 (81.7)	153.9 (50.1)	6.46	<0.001	45.7, 94.7	13.52 (<0.001)	46.64 (<0.001)	<0.001
	CS	PE						
	146.4 (44.9)	153.9 (50.1)	-0.86	0.390	-24.7, 9.7			
	M700	PE						
	184.3 (86.2)	153.9 (50.1)	2.36	0.020	4.9, 55.9			
	M500	PE						
	202.8 (71.1)	153.9 (50.1)	4.35	<0.001	26.7, 71.2			<0.001

The difference in marginal fit determined by mean marginal gap measurements of prostheses fabricated from digital impressions is shown in Table 4. One-way ANOVA revealed no significant difference in average marginal gap measurements among the digital impression methods for single crowns (p = 0.146). However, for the three-unit prostheses, the difference in average marginal gap measurements was statistically significant (p<0.001). Tukey’s post hoc test revealed significant differences between the CS and DW (t = -5.85, p<0.001), M700 and DW (t = -3, p = 0.016), M700 and CS (t = 2.85, p = 0.024), and M500 and CS (t = 4.25, p<0.001).

**Table 4:** Summary of the differences in the mean marginal gap measurements of FDP frameworks fabricated from digital impressions FDP: fixed dental prostheses; DW: Dental Wings; CS: Carestream 3600; M700: Medit i700; M500: Medit i500

Crown type	Impression method and measurements (µm, sd in parenthesis)		t value	p-value	95% CI	ANOVA F statistic (p value)	Tukey’s post hoc t (p-value)
Single-crown (n=120)	DW	CS					
	185 (63.7)	177.1 (81.3)	0.42	0.678	-29.3,45.6	1.83 (0.146)	
	DW	M700					
	185 (63.7)	157.9 (48.7)	1.85	0.070	-2.3,56.3		
	DW	M500					
	185 (63.7)	195.9 (61.7)	-0.67	0.502	-43.3,21.5		
	CS	M700					
	177.1 (81.3)	157.9 (48.7)	1.11	0.272	-15.5,53.8		
	CS	M500					
	177.1 (81.3)	195.9 (61.7)	-1.01	0.317	-56.1,18.5		
	M700	M500					
	157.9 (48.7)	195.9 (61.7)	-2.64	0.011	-66.7,-9.2		
Three-unit FDP (n=240)	DW	CS					
	224.2 (81.7)	146.4 (44.9)	6.46	<0.001	53.9,101.6	12.32 (<0.001)	-5.85 (<0.001)
	DW	M700					
	224.2 (81.7)	184.3 (86.2)	2.59	0.011	9.5,70.2		-3.0 (0.016)
	DW	M500					
	224.2 (81.7)	202.8 (71.1)	1.52	0.129	-6.3,49.0		
	CS	M700					
	146.4 (44.9)	184.3 (86.2)	-3.02	0.003	-62.8, -13.1		2.85 (0.024)
	CS	M500					
	146.4 (44.9)	202.8 (71.1)	-5.19	<0.001	-77.9,-34.9		4.25 (<0.001)
	M700	M500					
	184.3 (86.2)	202.8 (71.1)	-1.28	0.202	-47.1,10.1		

## Discussion

The marginal fit of FDP is an important determinant of the treatment outcome. Poor margins may act as conduits for microleakage and plaque retention, compromise pulpal and gingival health, and ultimately result in treatment failure. In this digital era of dentistry, evidence-based decisions are critical for best practices in patient care, especially in resource-strained settings often encountered in low- and middle-income countries. Yet the prohibitive cost of digital systems remains an obstacle to the provision of state-of-the-art oral healthcare. Various authors have proposed marginal gap measurements of 75, 100, 160, and even 200µm to be within the acceptable range [29]. Moreover, a marginal discrepancy of up to 50µm has also been suggested as an acceptable limit [30]. In this study, a marginal fit for crowns as determined by mean marginal gap measurements of 150µm or less was deemed acceptable from a clinical standpoint. In the current study, conventional impressions produced single-crown and three-unit FDP frameworks with mean marginal gap measurements within the acceptable limit of 150µm. Furthermore, there was no statistically significant difference in the mean marginal gap measurements of the single-crown and three-unit FDPs produced from conventional impressions. Polyvinyl siloxane and polyether are presently the recommended conventional impression materials for prosthodontic treatment due to their accuracy, fine detail reproduction, and dimensional stability. Polyether has the additional advantage of being hydrophilic and hence ideal in a moist oral environment [15, 31]. The acceptable marginal gap measurements of FDP frameworks produced from polyether in this study confirmed the suitability of polyether as a control for comparison with newer digital systems.

Several digital scanning techniques are currently available [1, 11, 32-37]; hence, in this study, four digital scanning systems commonly used in the Kenyan market were selected for evaluation. All the scanners produced single-crown and three-unit FDPs with mean marginal gap measurements that were significantly greater than the acceptable measurement of 150 µm, except those for the three-unit FDPs fabricated using the CS 3600 digital scanner. Moreover, the measurements for the three-unit prostheses, except for those produced using CS 3600, were consistently greater than those for the single crown. Nonetheless, when the standard deviations of the means were considered, all the scanners demonstrated the potential to produce FDPs with marginal gap measurements within the acceptable limit of 150µm.

Dental Wings and CS 3600 are some of the latest powder-free intraoral scanners that enable dental professionals to scan patients’ teeth to create color 3D images [1]. Like the CEREC BlueCam, CS is a click-and-point system requiring the user to keep the wand still during capture. The Medit500 is marketed as an 'easy entry into digital dentistry' due to its affordability compared to the cost of other systems. However, unlike systems such as CEREC, Dental Wings, and Carestream, which offer a complete digital workflow, the M500 is supplied only as a laboratory scanner. The intraoral scanner from the same company is M700, with specifications superior to those of the laboratory scanner. Studies that are available on the manufacturer’s website [24, 26] imply that even though both scanners yield results that are still within the acceptable range in terms of marginal fit, M700 has superior qualities in terms of marginal fit for both single-crown and three-unit FDPs. This finding was similarly demonstrated in this study. Medit digital scanners are compatible with a wide range of applications that use software that allows the import of STL, polygon (PLY), and other object information (OBJ) file formats. All these digital systems promise clinician and patient convenience by using computerized, laser, optical, and miniaturization technologies to synchronize intraoral and laboratory processes to enable single-appointment fixed prostheses [1, 38].

A comparison of the marginal gap measurements of single-crown and three-unit FDPs fabricated from conventional and digital impressions in vitro revealed significant differences in both types of prostheses. The marginal fit of the three-unit prosthesis was significantly different among the five impression methods and the four digital methods. Moreover, Dunn’s post hoc test showed that the difference in overall fit between the conventional and digital impression methods was statistically significant between the polyether method and the three digital methods, except CS. Tukey’s post hoc test showed that the difference in marginal fit among the digital impression methods was statistically significant between CS and the other three digital methods (DW, M700, and M500) and between DW and M700. The marginal fit of the single crown prosthesis was significantly different among the five impression methods but not among the four digital methods. Dunn’s post hoc test showed that the difference in marginal fit between the conventional and digital impression methods was statistically significant between the polyether and two digital methods (DW and M500) but not between the CS or M700 methods.

While the findings of this in vitro study and other related ones demonstrate that both digital and conventional impression techniques can be used to accurately produce prostheses with an acceptable marginal fit [33], the outcome in terms of prosthesis span may be dependent on the digital system and user experience. Admittedly, in vivo, the presence of saliva, difficulties linked to the operator and patients, and challenges related to the prosthesis and the laboratories can lead to notable differences between the different techniques. Nonetheless, it has been shown that for single crowns, digital impressions produce restorations with a better fit than conventional impressions [36]. These findings were in tandem with the results observed for M700 but contrary to those for CS 3600 in the present study.

We acknowledge that the minor adjustments to some of the prostheses while fitting them onto the typodont model could have altered the measurements, hence a limitation of this study; nonetheless, this was done to very few of the specimens and was mitigated by having an adequate number of specimens for each type of prostheses and impression technique. We also acknowledge that the typodont teeth and model may be unstable and could have been a source of error during impression-making; however, we ensured that the teeth were secured tightly on the model using screws and that impressions were taken on a stable model on a dental laboratory bench. Further, there are more reliable methods of marginal gap measurements, such as stereomicroscope or scanning electron microscopy, but this was unavailable in a resource-strained setting such as where this study was conducted.

## Conclusions

Within the limitations of this study and considering the standard deviations of the mean marginal gap measurements, it was concluded that conventional impressions produced FDPs within clinically acceptable marginal gap limits of approximately 150µm for both single-crown and three-unit FDPs. In the digital category, CS produced FDPs within clinically acceptable marginal gap limits of approximately 150µm for three-unit fixed dental prostheses and M700 for single-crown prostheses. Statistically significant differences were observed between the conventional and digital impression techniques. For the three-unit FDPs, this difference was between polyether and all the other digital methods except for CS, while among the digital systems, the differences were between CS and the other three digital techniques, as well as between DW and M700. For the single-crown FDPs, the difference was statistically significant between polyether and DW and M500.

It is recommended that, within reasonable limits, both digital and conventional impression methods are accurate when fabricating FDPs. Among the impression techniques evaluated in this study, conventional impressions are suitable for producing accurate short- and long-span FDPs; furthermore, CS 3600 may be better for intraoral scanning of multiple or three-unit FDPs, while M700 may be better for intraoral scanning for single-crown FDPs. Nonetheless, intraoral scanners and laboratory scanners should not be used interchangeably.
